# Supplementary material for: Rho Kinase regulates neutrophil NET formation that is involved in UVB-induced skin inflammation
Source: Theranostics. 2022 Feb 7;12(5):2133–49. doi: 10.7150/thno.66457 (PMC8899566; doi:10.7150/thno.66457)
Supplement: Supplementary file 1 — Supplementary figures. [file thnov12p2133s1.pdf]

Supplemental Materials

Rho Kinase regulates neutrophil NET formation that is involved in UVB-induced skin inflammation

Minghui Li[1,2,3†], Xing Lyu[1,2,3†], James Liao[4], Victoria P. Werth[1,2], Ming-Lin Liu[1,2]

[1] Corporal Michael J. Crescenz VAMC, Philadelphia, PA, 19104, USA

[2] Department of Dermatology, Perelman School of Medicine, University of Pennsylvania, Philadelphia, PA, 19104, USA

[3] Department of Rheumatology and Immunology, Tianjin Medical University General Hospital, Tianjin 300052, China

[4] Division of Cardiology, Department of Medicine, University of Chicago, Chicago, IL, USA

[†] Contribution equally

Running title: ***Li et al. Rho kinase and NETosis***

Key words: ***Rho kinase, Neutrophil, NETosis, UVB, Skin inflammation.***

Correspondence to Ming-Lin Liu, MBBS, PhD, Department of Dermatology, Perelman School of Medicine, University of Pennsylvania, and Philadelphia Veterans Administration Medical center, Philadelphia, PA 19104,
eMAIL: lium1@pennmedicine.upenn.edu,

Supplemental Figures

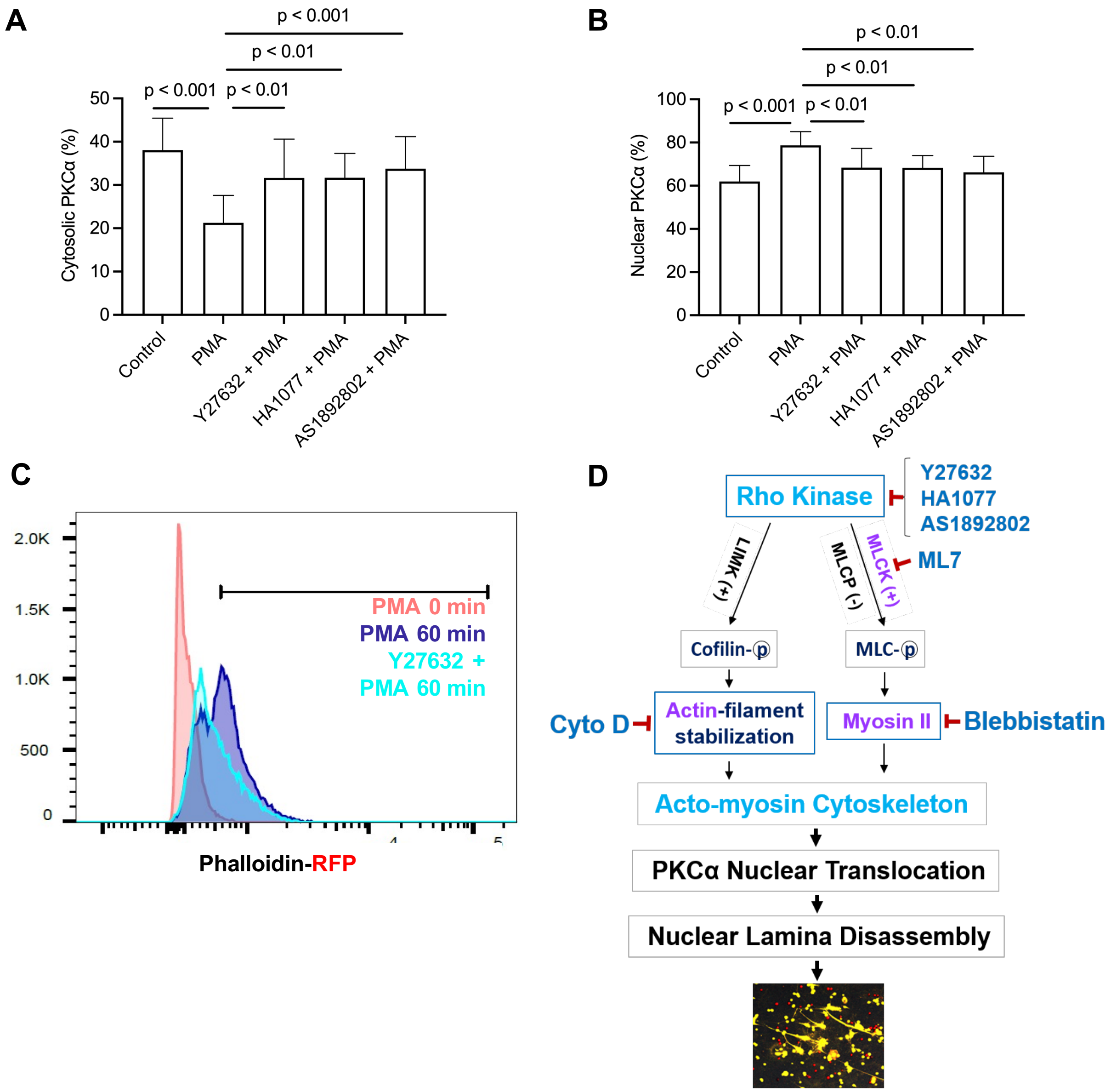

Figure S1. (**A,B**) Summary analyses of time course study of PKCα distribution in cytosolic (A) vs nucleus (B) in dPMNs treated by 50 nM PMA for 60 min, then stained for PKCα (primary anti-PKCα, and PE-labeled secondary antibody) and lamin B (primary anti-lamin B, and FITC-labeled secondary antibody), following by confocal fluorescent microscopy, and Image J analyses. (**C**) Representative analysis of F-actin polymerization detected by phalloidin-RFP with flow cytometry in dPMNs stimulated by 50 nM PMA for 60 min without or with pretreatment by 50 µM Y27632, ROCK inhibitors for 30 min. (**D**) Schematic illustration of the involvement of actin cytoskeleton, actomyosin cytoskeletal networks, their upstream signaling pathways, and ROCK, as well as their corresponding inhibitors in NETosis. Panels A,B were summary analyses of at least 10 cells of each condition from three independent experiments. ANOVA analysis with post hoc Student-Newman Keuls test were conducted for these sample sets with normal distribution.

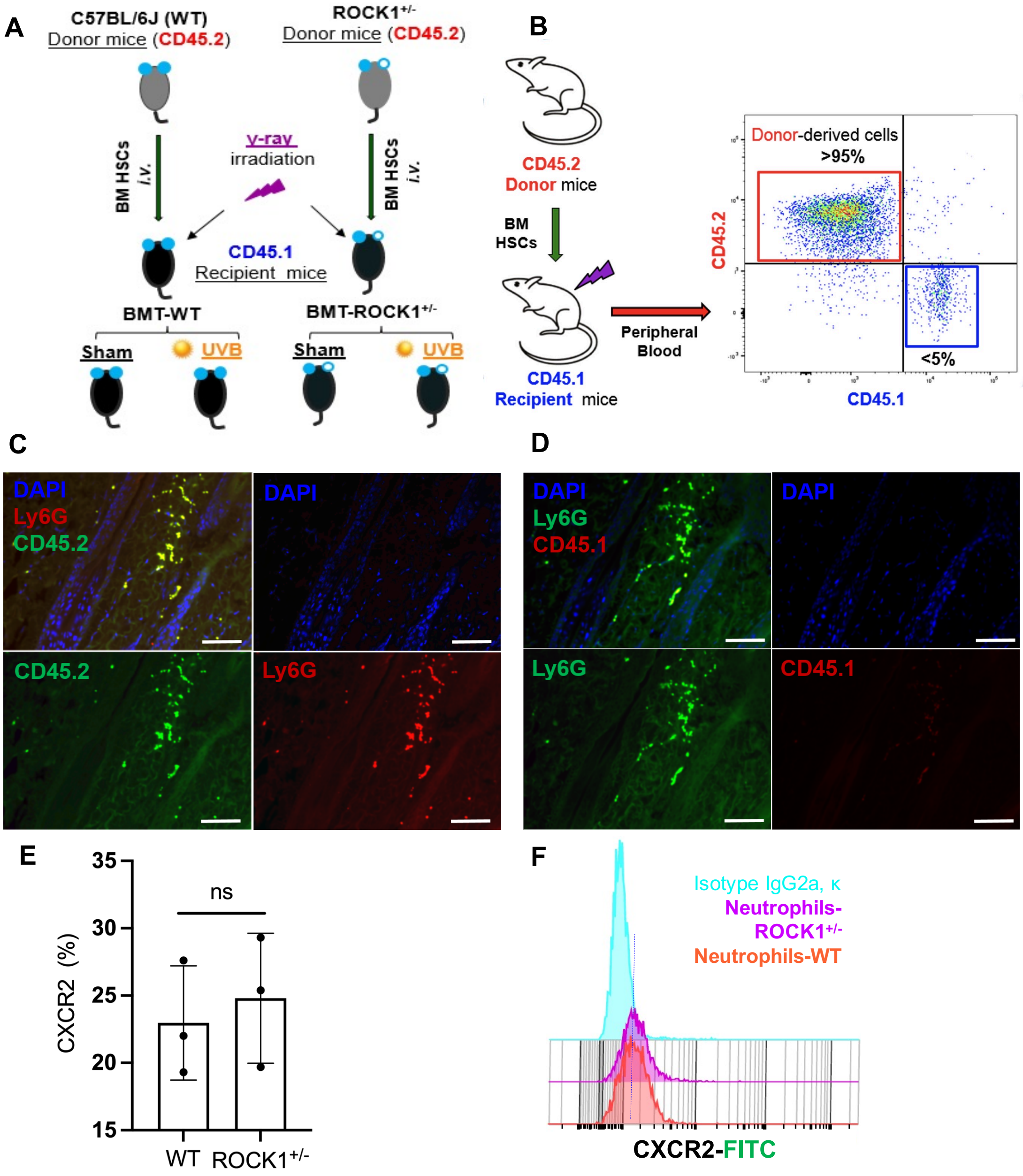

Figure S2. (**A**) Experimental design of the BMT-WT and BMT-ROCK1$^{+/-}$ mice that were irradiated without or with UVB. (**B**) Representative dot plots of flow cytometry analysis of all BMT experimental mice for detection of reconstitution rate of donor-derived cells (CD45.2) in peripheral blood of recipient mice (CD45.1). (**C,D**) Representative images of immunohistochemistry staining of skin lesions from UVB-irradiated CD45.1 recipient mice with BMT of HSCs from WT donors, stained by antibodies against Ly6G (PE) and CD45.2 (FITC) (C), or antibodies against CD45.1 (PE) and LY6G (FITC) (D), and DAPI for DNA. (**E,F**) Summary analyses and representative histograms of flow cytometry analyses of CXCR2 expression in BM neutrophils from WT vs ROCK1$^{+/-}$ mice, stained by fluorescent conjugated antibodies against mouse Ly6G (PE), CXCR2 (FITC) and its isotype control IgG2a,κ (FITC). Un-paired student's t-test was conducted (E), and statistical significance was considered at a level of P-value < 0.05.

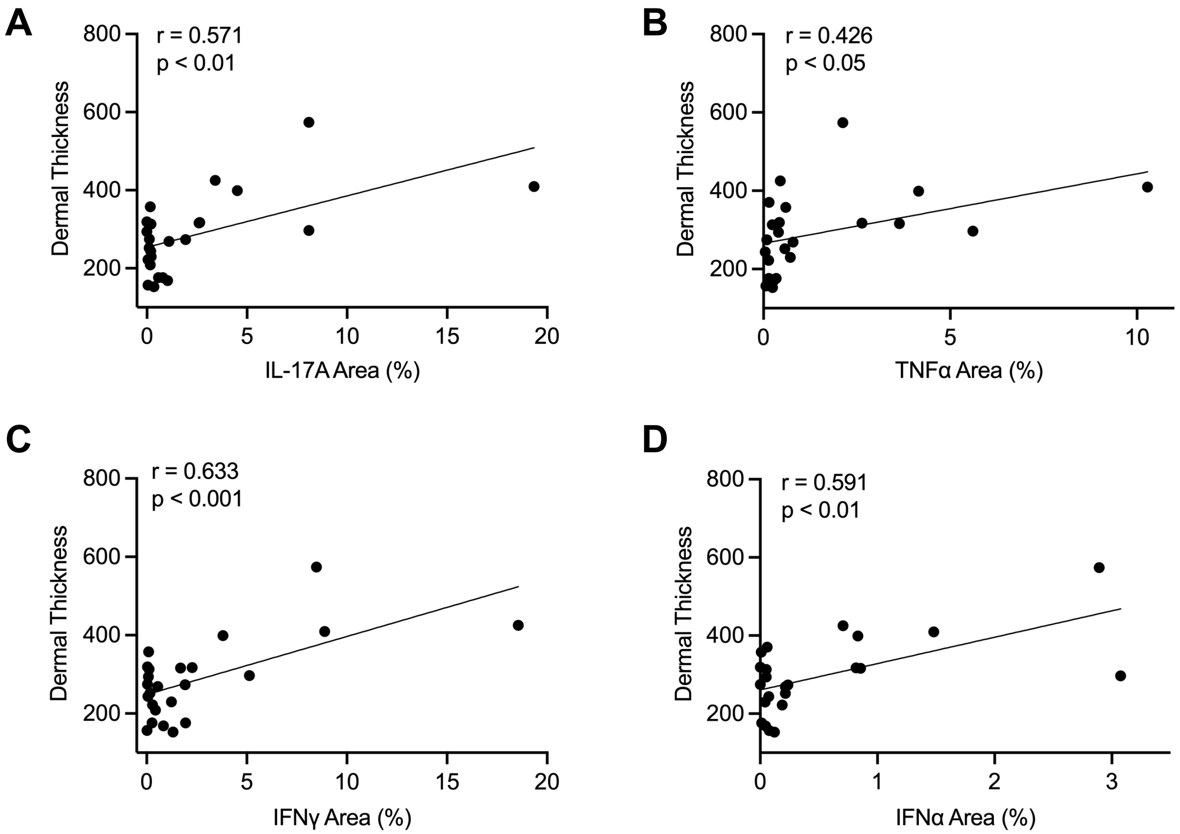

Figure S3. (**A-D**) Correlation between NET-associated IL-17A (Area %), TNFα, IFNγ, or IFNα and dermal thickness in all groups of BMT experimental mice. Pearson correlation analysis was conducted to analyze correlation between NET-associated cytokines and dermal skin thickness.

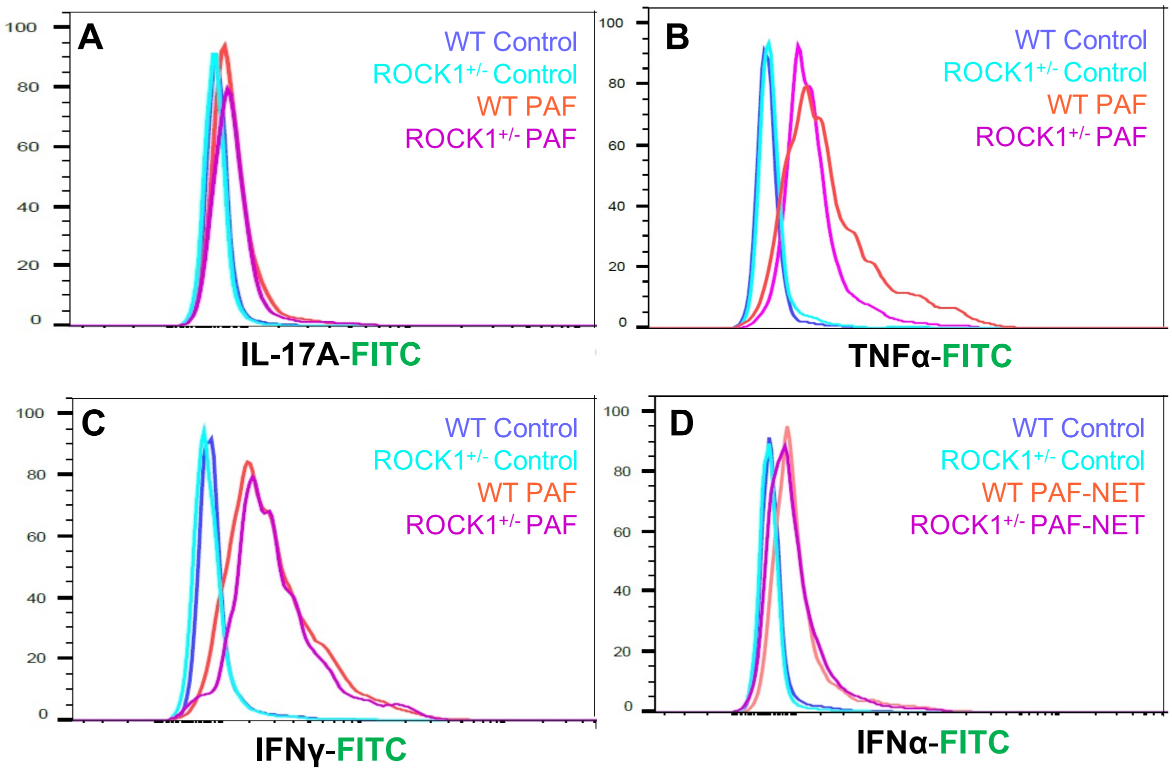

Figure S4. (**A-D**) Representative histograms of flow cytometry analyses of BM neutrophils, from ROCK1$^{+/-}$ mice or their WT littermates, which were treated without or with 10 μM PAF (for IFNα induction in panel D, treatment with PAF and NETs isolated from cultured neutrophils of MRL/lpr mice) for 20h, then fixation and staining with PE-conjugated anti-mouse Ly6G, and FITC labeled anti-mouse IL-17A, TNFα, IFNγ, or IFNα, following by flow cytometry analyses (Summary data, see Figure 6A,D,G,J).
